# Identifying clinical features and molecular characteristics of the endometrial clear cell carcinoma

**DOI:** 10.3389/fonc.2023.1286176

**Published:** 2023-11-17

**Authors:** Yuhan Cai, Qin Han, Hongyan Guo

**Affiliations:** Department of Gynecology, Peking University Third Hospital, Beijing, China

**Keywords:** endometrial clear cell carcinoma, molecular characteristics, TCGA, HER-2, TAF1

## Abstract

**Objective:**

The aim of this study was to verify TCGA subtypes in endometrial clear cell carcinoma (ECCC) and determine their clinical and molecular characteristics.

**Methods:**

We summarized and compared the clinical features of 28 clear cell carcinoma and 112 endometrioid carcinoma patients. Of the 28 ECCCs, 19 underwent TCGA classification, and other markers (ER, PR, ARID1A, ARIB1B, TAF1, and HER-2) were also detected by IHC, and outcomes were assessed.

**Results:**

Compared to endometrioid carcinoma, ECCC had an older age of onset (median age, 64.5 years, range 31–81 years), higher rate of myometrial invasion (42.8% vs. 21.5% in endometrioid carcinoma), LVSI (33% vs. 16%), and more advanced FIGO stage. Among the ECCCs, LVSI was a poor prognostic factor. TCGA classification was performed for 19 ECCCs: two POLEmut cases (10.5%), three MMRd (15.8%), 11 p53wt (57.9%), and three p53abn (15.8%). Of the 19 ECCCs, six (31.6%) showed HER-2 positive expression, and eight (42.1%) had TAF1 expression loss. ECCCs possessed HER-2 and TAF1 expression had worse outcomes.

**Conclusion:**

Our study summarized the clinical features of ECCC. The outcomes of patients with ECCC with TCGA subtypes differed from those of patients with endometrioid carcinoma. HER-2 and TAF1 may be new prognostic factors.

## Introduction

Endometrial carcinoma (EC) is one of the most common gynecological malignancies in the China (1). A total of 71,100 new EC cases are diagnosed, accounting for 4% of all new cancers diagnosed in women, making EC the ninth most common cancer among women. Endometrial clear cell carcinoma (ECCC) is a rare type of EC. Only 1%–5% of ECs are diagnosed as ECCC (2, 3).

According to the traditional dualistic model of EC proposed by Bokhman, ECCC is classified as “type II” (4), which is estrogen independent. However, the dualistic model cannot explain the heterogeneity, inconsistency between pathological and molecular types, and clinical behavior (5, 6).

The Cancer Genome Atlas (TCGA) (7) Research Network introduced four molecular subtypes of EC: POLE/ultramutated (POLE), microsatellite instability/hypermutated (MSI), copy number low/TP53 wild-type (CN-L), and copy number high/TP53 mutant (CN-H). However, this study only included endometrioid and serous carcinomas, and the relationship between ECCC and TCGA classification is less well understood. DeLair (8) first proved that ECCC can be divided into four subtypes. A meta-analysis revealed the distribution of the four ECCC subtypes (9). The most prevalent subgroup was CNH (42.5%), followed by CNL (40.9%), MSI (9.8%), and the less common was POLE (3.8%).

However, other studies have reported different results. Hoang (10) only found one POLE mutation out of 63 ECCCs and Baniak (2) only found six out of 37. Notably, none of these mutations were hotspot mutations. No cases were MMR-deficient in the study by Baniak et al. In contrast, another study reported no cases of aberrant p53 expression and a relatively high incidence of MSI (33.3%) (11).

Therefore, further studies are necessary to help us understand whether ECCC can be divided into four subtypes according to TCGA molecular signatures and whether ECCC has unique molecular characteristics. In this study, we attempted to verify TCGA subtypes in ECCC and determine their clinical features and molecular diversity.

## Materials and methods

### Case selection

We selected patients diagnosed with ECCC (n = 28) from the files of the Department of OBGYN at Peking University Third Hospital between 2009 and 2020. All 28 patients had clinical data and 19 of them had both slides and blocks available. We also randomly selected 112 cases of endometrioid carcinoma during the same period to compare the clinical characteristics with ECCC. Both ECCC and endometrioid carcinoma cases were independently reviewed by two gynecological pathologists. Clinical characteristics such as age, BMI, serum tumor biomarker, FIGO stage, LVSI status, myometrial invasion, and therapy for both ECCC and endometrioid carcinoma were collected and compared. This study was approved by the Institutional Review Board.

### TCGA classification

A diagnostic algorithm proposed by Murali was used. POLE hotspot mutation, IHC for DNA mismatch repair (MMR) proteins (MLH1, PMS2, MSH2, and MSH6), and p53 were performed on the 19 ECCC cases. POLE hotspot mutation, MMR deficiency, p53 wild-type and p53 mutation corresponded to the POLE, MSI, CNL and CNH TCGA classifications, respectively.

### Additional immunohistochemistry

Immunohistochemistry (IHC) for ER, PR, HER-2, PIK3CA, ARID1A, ARID1B, and TAF1 was also performed on 19 ECCC cases, as previously described.

### Statistical analysis

Fisher’s exact test and the t-test were used for categorical and continuous data, respectively. The association between molecular subtype and overall survival was analyzed, and the Kaplan–Meier method was applied to the survival curves. Statistical significance was set at P value <0.05.

## Results

### Clinical characteristics of ECCC and endometrioid carcinoma

There were 28 cases of ECCC and 112 cases of endometrioid carcinoma with confirmed diagnoses in our study. The clinical characteristics of the two groups are detailed in **Table 1**. The median age at diagnosis was 64.5 years (range, 31–81 years) in the ECCC group and 55 years (range, 25–83 years) in endometrioid carcinoma group, and the difference between these two groups was statistically significant (P = 0.000). The median BMI were 23 kg/m^2^ (range, 18 kg/m^2^–40.2 kg/m^2^) and 25.23 kg/m^2^ (range, 18.59 kg/m^2^–42.61 kg/m^2^) respectively. It seemed that patients in the ECCC group had a lower BMI than their counterparts, but the P-value was 0.083. No difference was found in the serum CA125 (P = 0.604) and CA199 (P = 0.542) levels. Myometrial invasion extending to the outer half was found in 42.8% (12/28) of the ECCC group and 21.5% (24/112) of the endometrioid group, indicating that ECCC was more likely to extend to the deeper myometrium (P = 0.02). The rate of LVSI in ECCC was higher than that carcinoma (33% vs. 16%, P = 0.044). Approximately 32% of patients with ECCC presented with advanced-stage disease, but only 10% in the endometrioid group (P = 0.007).

**Table 1:** Descriptive statistics of clear cell carcinoma and endometrioid carcinoma for clinical characteristics.

	Clear Cell Carcinoma	Endometrioid Carcinoma	P-value
BMI			
Median (IQR)	23 (21.8175–27.4924)	25.23 (22.765–27.8075)	0.083
Age			
Median (IQR)	64.5 (58–74.5)	55 (49–61)	0.000
CA125			
Median (IQR)	18.25 (13.13–42.68)	18.01 (11.92–30.4)	0.604
CA199			
Median (IQR)	17.75 (10.35–33.64)	13.905 (8.5075–28.775)	0.542
High blood pressure			0.602
Yes	12 (42.8%)	42 (37.5%)	
No	16 (57.2%)	70 (62.5%)	
Diabetes Mellitus			0.751
Yes	5 (17.8%)	23 (20.5%)	
No	23 (82.2%)	89 (79.5%)	
Stage			0.007
I	18 (65%)	99 (88%)	
II	1 (3%)	2 (2%)	
III	4 (14%)	8 (7%)	
IV	5 (18%)	3 (3%)	
LVSI			0.044
Yes	9 (33.0%)	18 (16.0%)	
No	18 (67.0%)	93 (84.0%)	
Myometrial invasion			0.02
≦̸1/2	16 (57.2%)	88 (78.5%)	
>1/2	12 (42.8%)	24 (21.5%)	

**Figure 1:**
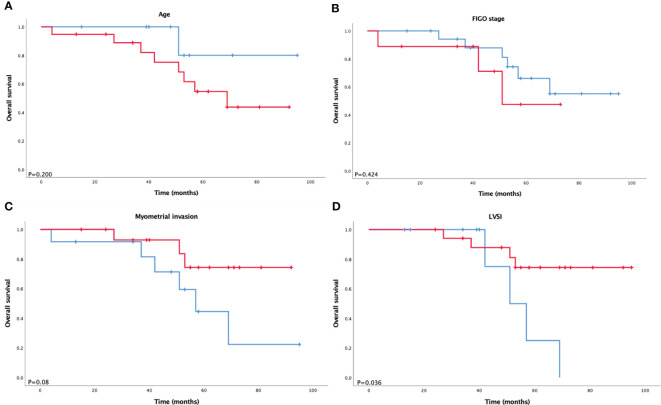
Kaplan–Meier survival analysis of overall survival according to **(A)** age, **(B)** FIGO stage, **(C)** myometrial invasion, and **(D)** LVSI in ECCC.

Overall survival (OS) analysis of patients with ECCC was also performed. The median follow-up period was 52 months (range, 4–95 months). Kaplan–Meier overall survival curves for ECCC patients stratified according to FIGO stage (stage I/II vs. stage III/IV), age (≤60 years vs. >60 years), LVSI status, and myometrial invasion are presented in **Figure 1**. There was a trend that worse outcomes were concerned with advanced stage, elder age, LVSI, and deep myometrial invasion but shorter overall survival was only significantly associated with LVSI (P = 0.036).

### TCGA classification of ECCC

**Figure 2:**
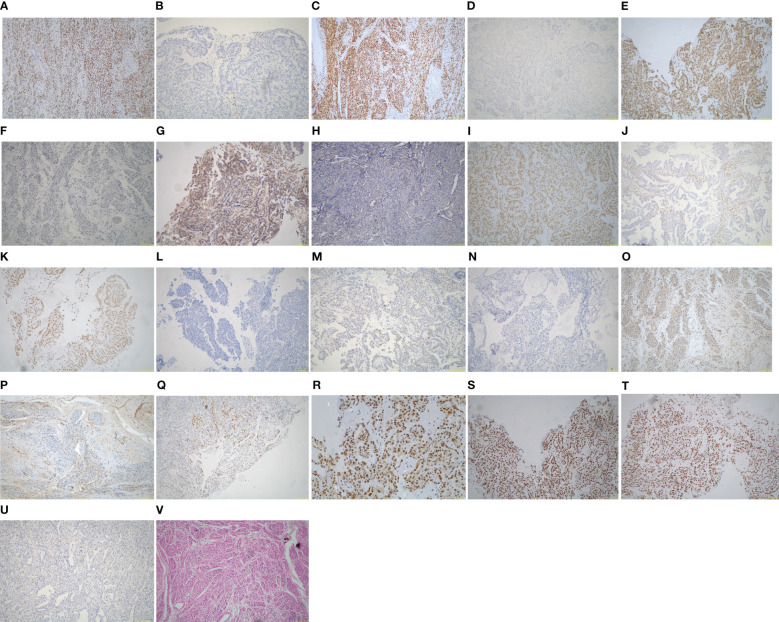
Immunohistochemistry and hematoxylin and eosin (H&E) of the ECCC patients (original magnification ×100). **(A)** ER positive. **(B)** ER negative. **(C)** PR positive. **(D)** PR negative. **(E)** HER-2 positive. **(F)** HER-2 negative. **(G)** PIK3CA positive. **(H)** PIK3CA negative. **(I)** ARID1A positive. **(J)** ARID1A negative. **(K)** ARID1B positive. **(L)** ARID1B negative. **(M)** p53 wild type. **(N)** p53 abnormal type. **(O)** TAF1 positive. **(P)** TAF1 negative. **(Q)** MLH1 positive. **(R)** PMS2 positive. **(S)** MSH2 positive. **(T)** MSH6 positive. **(U)** MSH6 negative. **(V)** H&E of ECCC.

**Figure 3:**
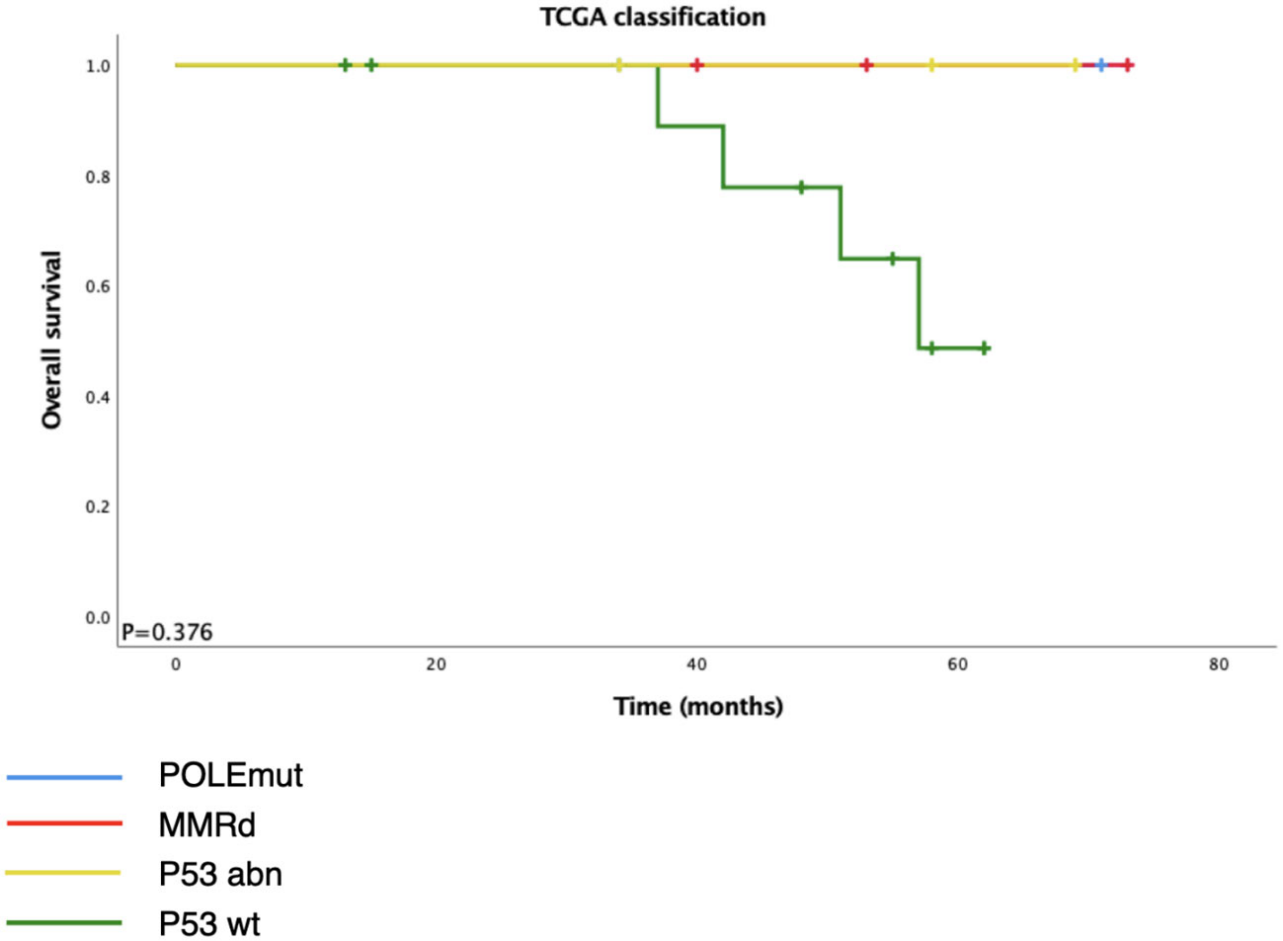
Kaplan–Meier survival analysis of overall survival according to TCGA classification.

Among the 28 ECCCs, 19 had tissues available for DNA extraction and IHC (**Table 2**, **Figure 2**). The molecular subtype distribution included two POLEmut cases (10.5%), three MMRd cases (15.8%), 11 p53wt cases (57.9%), and three p53abn cases (15.8%). Of the three MMRd cases, one had a family history of colorectal cancer and endometrial cancer and was considered a Lynch syndrome-related tumor. Kaplan–Meier survival analysis of TCGA classification is shown in **Figure 3**. POLE and MMRd appeared to have better outcomes than p53wt; however, p53abn appeared to have a better prognosis (P = 0.376).

**Table 2:** TCGA classification and immunohistochemistry information of ECCC.

Case ID	ER	PR	HER-2	TCGA	PIK3CA	ARID1A	ARID1B	TAF1	Survival condition	Follow up month
1	*Negative*	Negative	Positive	p53wt	Positive	Positive	Positive	Positive	Alive	58
2	Negative	Negative	Positive	p53wt	Negative	Positive	Positive	Positive	Died	51
3	Negative	Negative	Positive	p53wt	Negative	Positive	Negative	Positive	Died	42
4	Negative	Negative	Positive	p53abn	Positive	Positive	Positive	Positive	Alive	69
5	Negative	Negative	Positive	p53wt	Positive	Positive	Positive	Positive	Died	37
6	Negative	Negative	Negative	MMRd	Negative	Positive	Positive	Negative	Alive	40
7	Negative	Negative	Negative	p53wt	Negative	Positive	Positive	Negative	Alive	13
8	Negative	Negative	Negative	MMRd	Positive	Negative	Positive	Positive	Alive	73
9	Negative	Negative	Negative	POLEmut	Positive	Positive	Positive	Positive	Alive	34
10	Positive	Negative	Negative	p53wt	Positive	Positive	Positive	Positive	Alive	48
11	Positive	Negative	Negative	p53wt	Positive	Positive	Positive	Negative	Alive	55
12	Negative	Negative	Negative	p53wt	Positive	Positive	Positive	Negative	Alive	62
13	Positive	Positive	Negative	POLEmut	Negative	Positive	Positive	Negative	Alive	71
14	Negative	Negative	Negative	p53wt	Negative	Positive	Positive	Positive	Alive	62
15	Negative	Negative	Negative	p53abn	Negative	Positive	Positive	Negative	Alive	58
16	Negative	Negative	Negative	p53abn	Negative	Positive	Positive	Negative	Alive	34
17	Negative	Negative	Negative	MMRd	Negative	Negative	Positive	Positive	Alive	53
18	Positive	Negative	Positive	p53wt	Positive	Positive	Positive	Negative	Alive	15
19	Negative	Negative	Negative	p53wt	Positive	Positive	Positive	Positive	Died	57

### Other immunohistochemistries of ECCC

The histological features of the 19 ECCCs are presented in **Table 1** and **Figure 2**. Four of the 19 ECCCs (21.1%) were ER-positive and only one ECCC (5.3%) was PR-positive. ARID1A loss was observed in two cases (10.5%) and one case had ARID1B loss of expression. Loss of PI3KCA occurring in 9/19 (47.4%) cases and TAF1 in 8/19 cases (42.1%), respectively. HER-2 negativity by IHC was observed in 13 cases (68.4%).

Further survival analysis showed that negative expression of HER-2 was associated with longer overall survival. Although the differences in the remaining markers were not statistically significant, we noticed that loss of TAF1 expression had an obvious tendency toward better prognosis (P = 0.146).

**Figure 4:**
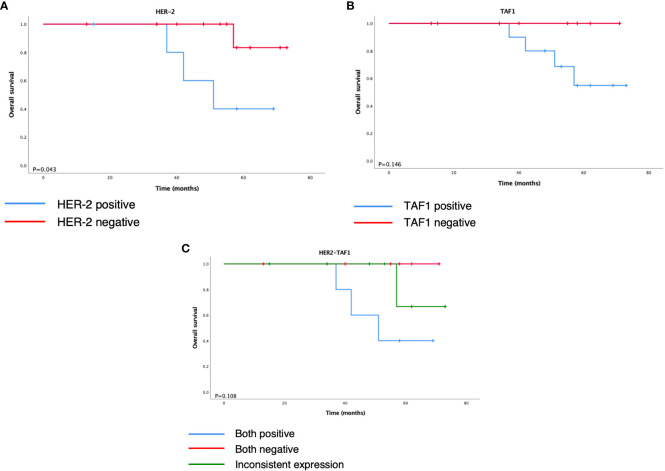
Kaplan–Meier survival analysis of overall survival according to **(A)** HER-2, **(B)** TAF1, and **(C)** HER2-TAF1.

As a result, the 19 cases were subdivided into three groups according to the status of HER-2 and TAF1: positive, negative, and inconsistent expression. Although the difference was not statistically significant (P = 0.108), the K–M curves demonstrated different trends of overall survival among the three groups (**Figure 4**). Both the positive expression groups showed a worse outcome, and the negative expression group showed the best prognosis among the three groups.

## Discussion

According to the dualistic model, type II patients tend to be older than their counterparts, usually diagnosed at the age of 66–68 years. The disease is estrogen-independent; therefore, lower BMI, high blood pressure, and diabetes rates are believed to exist. Type II patients are likely to have LVSI, deep myometrial invasion, and an advanced FIGO stage (1, 12–14). Similarly, in our study, compared with endometrioid carcinoma, ECCC patients were older (median age, 64.5 years), with more advanced stage (III–IV) in 32%, LVSI in 33%, and deep myometrial invasion in 42.8%. BMI, high blood pressure, diabetes, and blood serum biomarkers were associated with ECCC and endometrioid carcinoma to a similar extent. One reason might be that the sample size of ECCC was small because we could recognize that the median BMI of ECCC was lower than that of endometrioid carcinoma, although the p-value was higher than 0.05. In addition, a study by Setiawan et al. (13) showed no difference between type I and type II in factors like diabetes. As confirmed by previous studies, advanced stage, older age at diagnosis, and deep myometrial invasion were significant prognostic factors. In our study, we observe different trends in outcomes in terms of these three factors. LVSI is a vital step in tumor metastasis and is an important prognostic factor in endometrial carcinoma. Abeler and Kjørstad (15) revealed that LVSI is an important prognosticator of ECCC. Conversely, other studies (16, 17) have found that LVSI has no statistically significant clinical value. To verify these findings, we studied the prognostic value of LVSI and demonstrated a significant correlation with a shorter OS (P = 0.036).

It is important to classify gynecological cancer patients according to their prognosis and tailor them to a more appropriate therapeutic and surveillance program (18). The Cancer Genome Atlas classifies endometrial carcinoma into four prognostic molecular subgroups; however, TCGA does not include endometrial clear cell carcinoma. According to a previous study (19), ECCC shows a wide overlap of features with both endometrioid and serous carcinomas at morphological, immunohistochemical, molecular, and prognostic levels. Several studies (2, 8, 10, 11, 20) have focused on the prevalence of TCGA subgroups in ECCC. Although some studies did not detect any POLE hotspot mutations and MMR deficiency, our results are in line with other studies that found a 10.5% frequency of POLE hotspot mutation and15.8% of MMR-D. Consistent with different reports (21), p53 was the most frequent type in our study (57.9%). Previous studies showed 42.5% p53abn in ECCC; it is noteworthy that the proportion of p53abn in our study was far less than that in previous studies (15.8%), and no cases of aberrant p53 expression were found in a cohort of 45 ECCC cases (11). Considering the heterogeneity of endometrial carcinoma and all the facts presented by these studies, we tentatively propose that ECCC may not completely fit the TCGA model and may have its own molecular characteristics.

HER-2 is an oncogene that is overexpressed in various types of tumors. Its overexpression may cause abnormal cell proliferation, inhibition of apoptosis, formation of tumor blood vessels and increased tumor invasiveness (22, 23). A study by Morrison (24) showed that higher HER-2 expression was correlated with higher grade and stage endometrial cancer and shorter disease-specific survival and progression-free survival. This study included nine ECCCs. Another study by Xiao et al. (25) found that HER-2 overexpression was significantly associated with higher clinical stage and lymph node metastasis, which were closely related to poor prognosis. However, in a study by Sarmadi (26), whose study recruited 74 endometrial cancer patients and nine of them had ECCC, there was no statistically significant difference between HER-2 expression and DFS or OS. They found that a high rate of myometrial invasion in ECCC was associated with HER-2 overexpression. Similarly, in our study, we detected positive expression of HER-2 in ECCC was associated with shorter overall survival (P = 0.043).

TATA-box-binding protein (TBP)-associated factor 1 (TAF1) is a key component of RNA polymerase II and is known to play a critical role in the regulation of cell growth and the cell cycle, whereas its role in cancer development is largely unknown (27–29). Some data suggest that TAF1 may contribute to the progression of hepatocellular carcinoma (HCC) and is significantly associated with OS in patients with HCC (30, 31), but other studies have shown that inactivation of TAF1 may be involved in tumorigenesis (29). As indicated in Oh’s study, the TAF1 frameshift mutation reduces cell death and contributes to the survival of gastric and colorectal cancer cells. In uterine cancers, TAF1 has been nominated as a candidate driver gene in uterine serous carcinoma, and somatic mutations of TAF1 have been found in some ECCCs (32). However, the relationship between the expression of TAF1 and prognosis in ECCC has not been confirmed. There was no statistically significant association between TAF1 expression and OS in ECCCs in our study (P = 0.146). However, we found no patients died in the TAF1 negative group during their follow-up and had a better prognosis than their counterparts.

Since ECCC may not completely fit the TCGA model and may have its own molecular characteristics, we subdivided the ECCCs into three groups according to our findings in HER-2 and TAF1: both positive expressions, both negative expressions, and inconsistent expression. Although the P-value was >0.05, which may partly be due to the limited sample size in this study, the three groups seemed to have obvious differences in OS. This study may provide a new molecular classification method. However, the association between HER-2 and TAF1 requires further exploration.

According to the results, it may be concluded that compared to endometrioid carcinoma, ECCC had an older age of onset, later FIGO stage, and deeper myometrial invasion. ECCC may not completely fit TCGA model. However, positive expression of HER-2 and TAF1 may affect prognosis, providing a novel possible molecular classification method and enlightening us to investigate the association between HER-2 and TAF1 in ECCC. Further studies with larger sample sizes are necessary to verify this conclusion.

## Data availability statement

The raw data supporting the conclusions of this article will be made available by the authors, without undue reservation.

## Ethics statement

The studies involving humans were approved by Peking University Third Hospital Institutional Review Board. The studies were conducted in accordance with the local legislation and institutional requirements. The human samples used in this study were acquired from paraffin-embedded tissue after surgery. Written informed consent for participation was not required from the participants or the participants’ legal guardians/next of kin in accordance with the national legislation and institutional requirements.

## Author contributions

YC: Data curation, Software, Writing – original draft. QH: Formal Analysis, Methodology, Writing – review & editing. HG: Funding acquisition, Methodology, Writing – review & editing.
